# Comprehensive behavioral analysis of RNG105 (Caprin1) heterozygous mice: Reduced social interaction and attenuated response to novelty

**DOI:** 10.1038/srep20775

**Published:** 2016-02-11

**Authors:** Rie Ohashi, Keizo Takao, Tsuyoshi Miyakawa, Nobuyuki Shiina

**Affiliations:** 1 Laboratory of Neuronal Cell Biology, National Institute for Basic Biology, Okazaki, Aichi, Japan; 2 Department of Basic Biology, SOKENDAI, Okazaki, Aichi, Japan; 3 Okazaki Institute for Integrative Bioscience, Okazaki, Aichi, Japan; 4 Section of Behavior Patterns, National Institute for Physical Science, Okazaki, Aichi, Japan; 5 Department of Physiology, SOKENDAI, Okazaki, Aichi, Japan; 6 Division of Animal Resources and Development, Life Science Research Center, University of Toyama, Toyama, Japan; 7 Division of Systems Medical Science, Institute for Comprehensive Medical Science, Fujita Health University, Toyoake, Aichi, Japan

## Abstract

RNG105 (also known as Caprin1) is a major RNA-binding protein in neuronal RNA granules, and is responsible for mRNA transport to dendrites and neuronal network formation. A recent study reported that a heterozygous mutation in the *Rng105* gene was found in an autism spectrum disorder (ASD) patient, but it remains unclear whether there is a causal relation between RNG105 deficiency and ASD. Here, we subjected *Rng105*^+/*−*^ mice to a comprehensive behavioral test battery, and revealed the influence of RNG105 deficiency on mouse behavior. *Rng105*^+/*−*^ mice exhibited a reduced sociality in a home cage and a weak preference for social novelty. Consistently, the *Rng105*^+/*−*^ mice also showed a weak preference for novel objects and novel place patterns. Furthermore, although the *Rng105*^+/*−*^ mice exhibited normal memory acquisition, they tended to have relative difficulty in reversal learning in the spatial reference tasks. These findings suggest that the RNG105 heterozygous knockout leads to a reduction in sociality, response to novelty and flexibility in learning, which are implicated in ASD-like behavior.

In neurons, mRNA transport and local translation in dendrites play crucial roles in long-lasting synaptic plasticity and higher-order brain functions^1^,^2^,^3^. RNA granules are key macromolecular complexes for dendritic mRNA transport and synaptic stimulation-dependent translational control^4^,^5^. Translational regulators and RNA-binding proteins including the RNA granule components are emerging as factors associated with neurodevelopmental disorder, intellectual disability and mental disorder, e.g., the mammalian target of the rapamycin complex 1 (mTORC1) signaling pathway in autism spectrum disorder (ASD), the fragile X mental retardation protein (FMRP) in fragile X syndrome, and disrupted-in-schizophrenia 1 (DISC1) in schizophrenia^6^,^7^,^8^,^9^.

RNA granule protein 105 (RNG105) (Caprin1) is a major RNA-binding protein in the RNA granules and most highly expressed in the brain^10^,^11^. RNG105 is responsible for the transport of specific mRNA from the soma to dendrites, and an RNG105 knockout in mice results in the reduced transport of mRNAs including Na^+^/K^+^ ATPase subunit mRNAs and a reduced expression of encoded proteins in dendrites^12^. RNG105 knockout impairs synapse formation on dendrites, development of dendrites, and neuronal network formation *in vitro*^12^, suggesting that RNG105 regulates the synaptic and neuronal network functions.

A recent study aiming to detect genetic variants in ASD by whole-genome sequencing reported that a heterozygous *de novo* nonsense mutation in the *Rng105* (*Caprin1*) gene was found in a patient with Asperger’s syndrome^13^. The patient’s intelligence quotient (IQ) was above average, but adaptive behavior was below average and sociability was delayed^13^. The results suggested that *Rng105* (*Caprin1*) is a candidate risk gene for ASD, but it remains unclear as to whether there is a causal relation between RNG105 deficiency and ASD.

In the present study, to investigate the influence of RNG105 deficiency on mouse behavior, we subjected RNG105 heterozygous (*Rng105*^+/*−*^) mice to a comprehensive behavioral test battery^14^,^15^. Social interaction and responses to novelty were reduced in *Rng105*^+/*−*^ mice. In addition, although *Rng105*^+/*−*^ mice exhibited normal memory acquisition, they tended to have relative difficulty in reversal learning. Furthermore, RNG105-deficient neurons showed a reduction in the AMPA glutamate receptor (AMPAR) cell surface distribution in dendrites, which has been reported in other ASD-like mutant mice and thought to be related with the neuropathology of ASD^16^,^17^,^18^. The behavioral test battery, together with the analysis of the AMPAR distribution, suggested that an RNG105 deficiency led to ASD-like behavior in terms of sociality, response to novelty and flexibility in learning.

## Results

### RNG105 mRNA and protein expression level was reduced in *Rng105* ^+/*−*^ mice

**Figure 1:**
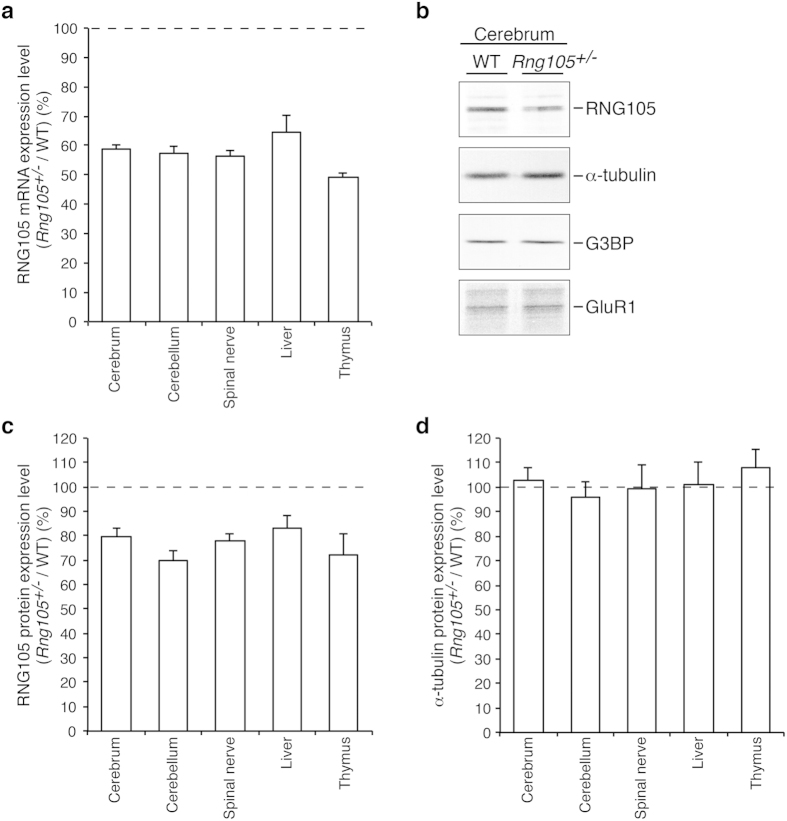
RNG105 mRNA and protein expression level in *Rng105*^+/−^ mice. (**a**) The ratio of RNG105 mRNA expression level in *Rng105*^+/−^ mice to that in wild-type mice. mRNA expression was measured by quantitative reverse transcription (RT)-PCR in each tissue from *Rng105*^+/−^ and wild-type mice. *n* = 4. (**b**) Western blotting for RNG105 and control proteins (α-tubulin, G3BP, GluR1) in the cerebrum from wild-type and *Rng105*^+/−^ mice. The same amount of extracts between wild-type and *Rng105*^+/−^ mice (40 μg per lane for RNG105, G3BP and GluR1; 0.25 μg per lane for α-tubulin) was loaded on each lane. (**c**,**d**) The ratio of the expression levels of RNG105 protein (**c**) and α-tubulin protein (**d**) in *Rng105*^+/−^ mice to that in wild-type mice. Protein expression was quantified by Western blotting of extracts from each tissue with an anti-RNG105 antibody and an anti-α-tubulin antibody (see Supplementary Fig. S1). *n* = 4. Data are presented as mean ± standard error of the mean (S.E.M).

We first examined the expression level of RNG105 mRNA and protein in tissues from *Rng105*^+/*−*^ mice. Brain and spinal nerve expressing high levels of RNG105, and liver and thymus expressing lower levels of RNG105^10^,^11^, were subjected to quantitative reverse transcription (RT)-PCR and Western blotting. In these tissues, RNG105 mRNA expression levels were reduced to about 50–60% in the *Rng105*^+/*−*^ mice compared with the wild-type mice (Fig. 1a). Western blotting of the brain indicated that the expression level of RNG105 protein, but not other control proteins, was reduced in the *Rng105*^+/*−*^ mice (Fig. 1b). Furthermore, quantification of RNG105 and α-tubulin as a control in brain, spinal nerve, liver and thymus revealed that RNG105, but not α-tubulin, was reduced in these tissues in the *Rng105*^+/*−*^ mice (Fig. 1c,d, see Supplementary Fig. S1). Although the reduction in the RNG105 protein levels was modest compared to the mRNA levels, the RNG105 protein expression levels were reduced to about 70-80% in the *Rng105*^+/*−*^ mice compared with the wild-type mice (Fig. 1c, see Supplementary Fig. S1).

### *Rng105* ^+/*−*^ mice showed abnormalities in social interaction

To evaluate the behavioral effects of RNG105 heterozygous knockout, we subjected the male *Rng105*^+/*−*^ mice and their wild-type littermates to a comprehensive battery of behavioral tests (Table 1). The physical characteristics of *Rng105*^+/*−*^ mice were comparable to those of wild-type mice (see Supplementary Fig. S2). The open-field activity (see Supplementary Fig. S3), motor skills and motor learning (see Supplementary Fig. S4) of *Rng105*^+/*−*^ mice were also comparable to those of wild-type mice except for the decrease in stride length in the gait analysis (see Supplementary Fig. S4d,j). There were no obvious differences between wild-type and *Rng105*^+/*−*^ mice in acoustic startle response/prepulse inhibition (see Supplementary Fig. S5b,c), anxiety-like behaviors (see Supplementary Fig. S6) or in the tail suspension test (see Supplementary Fig. S7c), although the *Rng105*^+/*−*^ mice showed increased pathological limb-clasping reflexes when suspended by the tail (see Supplementary Fig. S4q), shorter latency to withdraw their paws from a hot plate (see Supplementary Fig. S5a) and shorter immobility time in the Porsolt forced swim test (see Supplementary Fig. S7a).

**Figure 2:**
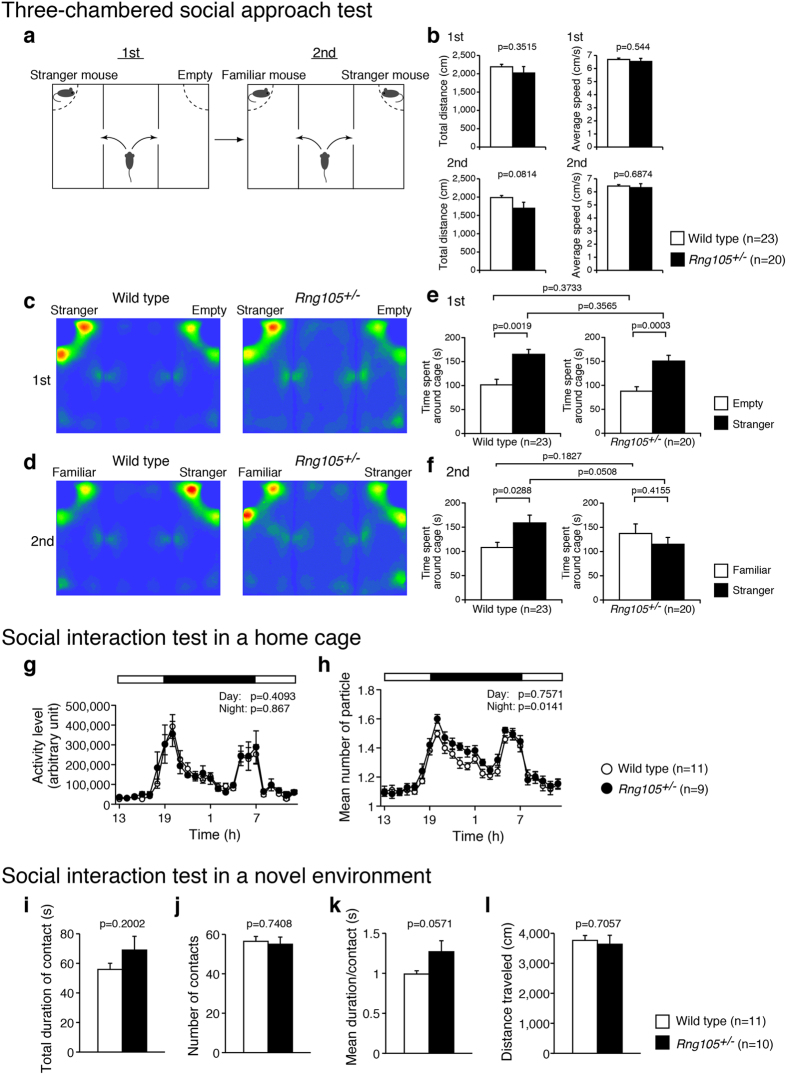
Social interaction abnormalities in *Rng105*^+/−^ mice. (**a**–**f**) Three-chambered social approach test. (**a**) Schematic diagram of the test. In the first session, one of the cages contains a stranger mouse. In the second session, the stranger mouse becomes familiar to the test mouse, and the other stranger mouse is put in the other cage. (**b**) Total distance traveled and the average locomotor speed in the first and the second sessions. (**c**,**d**) Heat maps showing the average traces of 23 wild-type mice (left panels) and 20 *Rng105*^+/−^ mice (right panels) in the first session (**c**) and the second session (**d**). (**e**,**f**) Time spent around either the empty cage or the stranger mouse cage in the first session (**e**) and either the familiar or stranger mouse cage in the second session (**f**). (**g**,**h**) Social interaction test in a home cage. White and black bars indicate lights on and off, respectively. The data are represented as the average of five days (day 3 to day 7) from a 7-day experiment. (**g**) Locomotor activity in the home cage test. (**h**) Social interaction of two mice as judged by the number of the particle. When the mice are apart from each other, the particle number is two, and the particle number is one when they are close to each other. Therefore, larger number of particle indicates less interactions. *n* = 11 and 9 pairs for wild-type and *Rng105*^+/−^ mice, respectively. (**i**–**l**) Social interaction test in a novel environment. Pairs of the same genotype mice were tested. (**i**) Total duration of contact with each other. (**j**) The number of contacts. (**k**) Mean duration per contact. (**l**) Total distance traveled. *n* = 11 and 10 pairs for wild-type and *Rng105*^+/−^ mice, respectively. Data are presented as mean ± S.E.M. P-values from one-way ANOVA (**b**, between different genotypes in **e** and **f**, and **i**-**l**), paired t-test (between the same genotypes in **e**,**f**) and two-way repeated measures ANOVA (**g**,**h**) are indicated.

Social interaction was examined using three different types of tests, i.e., the three-chambered social approach test, social interaction test in a home cage, and social interaction test in a novel environment. In the three-chambered social approach test, sociability and preference for social novelty were investigated (Fig. 2a). There were no significant differences in general activity such as total distance traveled or average locomotion speed between wild-type and *Rng105*^+/*−*^ mice (Fig. 2b). In the first session of this test, sociability, i.e., whether test mice show a preference for a cage with a stranger mouse to an empty cage, was tested. Both the wild-type and the *Rng105*^+/*−*^ mice spent a significantly longer time around the cage with a stranger mouse than the empty cage, and there were no significant differences between the genotypes (Fig. 2c,e). In the second session, social novelty, i.e., whether test mice show a preference for a cage with a stranger mouse to a cage with a familiar mouse, was tested. In contrast to the first session, the wild-type and the *Rng105*^+/*−*^ mice showed different preferences: the wild-type mice spent a significantly longer time around the cage with a stranger mouse than with a familiar mouse, but the *Rng105*^+/*−*^ mice spent an equivalent amount of time between the two cages (Fig. 2d,f). These results suggested that social interaction with a novel mouse was normal, but that there was a lack of preference for a novel mouse over a familiar mouse in *Rng105*^+/*−*^ mice.

A further test was the social interaction test in a home cage. In this test, pairs of the same genotype mice, which were strangers to each other, were put into a home cage for seven days. Locomotor activity and social interaction in the cage were measured and averaged from day 3 to day 7, when the mice were familiar with each other. There was no significant difference in the locomotor activity either during the day (7:00–19:00) or night (19:00–7:00) between wild-type and *Rng105*^+/*−*^ mice (Fig. 2g). In contrast, although the social interaction, which was judged by the mean number of particles, of the *Rng105*^+/*−*^ mice during the day was comparable to that of the wild-type mice, the social interaction of the *Rng105*^+/*−*^ mice was significantly less than wild-type mice during the night (Fig. 2h). In this test, the sample size was smaller than other tests because pairs of mice were used. However, a power analysis calculated the sample size (n) necessary to detect an effect to be n = 8.5 for each genotype with the parameters: effect size = 0.25, α = 0.05, power = 0.95 (recommendation values) and correlation among repeated measures = 0.96 (calculated from the data), suggesting that the sample size in this test (n = 11, 9) was effective to detect differences between genotypes^19^. Although replication of this behavioral test is needed in the future to increase the sample size and reduce the possibility that outlier mice could have large influence on the result, the present results suggested that the social interaction of familiar mice during an active period was reduced in the *Rng105*^+/*−*^ mice.

The other test was the social interaction test in a novel environment. In this test, two mice with the same genotype, which were strangers to each other, were put in a square box for 10 min. Although no statistically significant differences were observed (Fig. 2i–l), the mean duration per contact of *Rng105*^+/*−*^ mice tended to be longer than that of wild-type mice (Fig. 2k). Considered together, the results from the three tests suggested that the social interaction of *Rng105*^+/*−*^ mice had changed compared to wild-type mice: increased contact duration with novel mice, reduced preference for novel mice over familiar mice, and reduced interaction with familiar mice in a home cage.

### Reduced responses to novelty in *Rng105* ^+/*−*^ mice

The suggested reduced preference for novel mice led to the supposition that *Rng105*^+/*−*^ mice could also have an altered preference for other novel situations. Therefore, we performed a novel object recognition test and a place recognition test.

**Figure 3:**
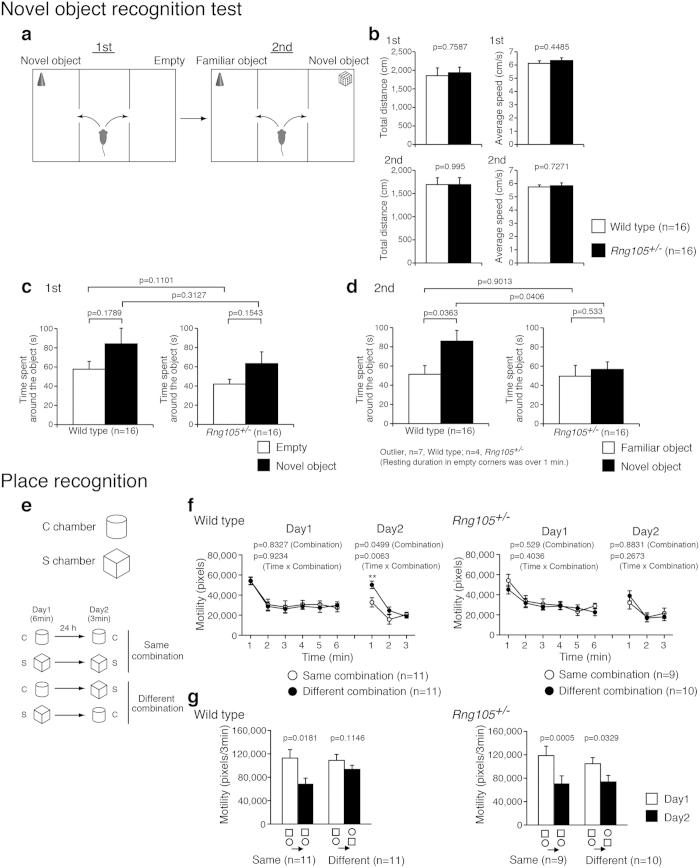
Response to novel objects and novel place patterns was reduced in *Rng105*^+/−^ mice. (**a**–**d**) Novel object recognition test. (**a**) Schematic diagram of the test. In the first session, an object is put in a corner of a chamber. In the second session, the object becomes familiar to the test mouse, and a different type of object is put in a corner of the other chamber. (**b**) Total distance traveled and the average locomotor speed in the first and the second sessions. (**c**,**d**) Time spent around either the empty corner or the novel object in the first session (**c**) and either the familiar or novel object in the second session (**d**). (**e**–**g**) Place recognition test. (**e**) Schematic diagram of the test. Mice were put into the C chamber (circle) or the S chamber (square) for 6 min on day 1. On day 2, they were put into the same chamber (same combination) or the other chamber (different combination) for 3 min. (**f**) Motility of wild-type mice (left panel) and *Rng105*^+/−^ mice (right panel) put into the same or different combination of the chambers on day 1 and day 2. (**g**) The sum of the motility in the first 3 minutes on day 1 (white bars) and 3 minutes on day 2 (black bars) for each group of mice. Data are presented as mean ± S.E.M. P-values from one-way ANOVA (**b**, between different genotypes in **c** and **d**), paired t-test (between the same genotypes in **c** and **d**, and **g**), and p-values for the main effect of the chamber combination and the interaction effect (time × chamber combination) in two-way repeated measures ANOVA (**f**) are indicated. **p < 0.01, one-way ANOVA at the same time point.

The experimental design of the novel object recognition test was similar to that of the three-chambered social interaction test except that different types of objects were used instead of stranger mice (Fig. 3a). There were no significant differences in general activity, such as total distance traveled or average locomotion speed, between the genotypes (Fig. 3b). In the first session, both wild-type and *Rng105*^+/*−*^ mice tended to spend a longer time around a novel object rather than the alternative empty side, although statistically it was not significant (Fig. 3c). The time *Rng105*^+/*−*^ mice spent around a novel object was comparable to wild-type mice (Fig. 3c). On the other hand, in the second session, the time spent around a novel object was significantly different between the wild-type and *Rng105*^+/*−*^ mice (Fig. 3d). Wild-type mice spent a longer time around a novel object than a familiar object, whereas *Rng105*^+/*−*^ mice spent almost the same amount of time between the novel and familiar objects (Fig. 3d). These results suggested that *Rng105*^+/*−*^ mice had a reduced preference for a novel object than a familiar object compared to wild-type mice, which was reminiscent of the reduced preference for social novelty in *Rng105*^+/*−*^ mice.

In the place recognition test, mice were put into a novel circle-type (C) or square-type (S) chamber to learn the place pattern on day 1. On day 2, the mice were put into the same chamber (same combination) or another chamber (different combination) to test adaptation for the chamber (Fig. 3e). We monitored the mouse motility, reduction of which suggests adaptation to a place. Wild-type mice showed a reduction in motility on day 2 when they were put into the same combinations of chambers (Fig. 3f,g, left panels). In contrast, they did not show a reduction in motility on day 2 when they were put into different combinations of chambers (Fig. 3f,g, left panels). In particular, the motility of wild-type mice put into the different chambers during the first one minute on day 2 was significantly higher than that of those put into the same chambers (Fig. 3f, left panel). On the other hand, *Rng105*^+/*−*^ mice showed significantly lower motility on day 2 than day 1 in both the same combinations and different combinations of chambers (Fig. 3f,g, right panels). Because the mice were divided into two groups and the sample size (n) for each group was small in this behavioral test, we also conducted nonparametric Mann-Whitney U test for the first one minute on day 2 (Fig. 3f). The result was p = 0.01 (U = 21) between the same and different combinations for wild-type mice and p > 0.05 (U = 34) for *Rng105*^+/*−*^ mice, which supported the above notion. Nonetheless, replication of this behavioral test is needed in the future to increase the sample size. These results suggested that wild-type mice discriminated novel places from familiar places, which was expressed as a higher motility in novel places than in familiar places, whereas *Rng105*^+/*−*^ mice did not show such different responses to familiar and novel places. The indistinguishable responses to familiar and novel places are likely to be consistent with the results wherein *Rng105*^+/*−*^ mice showed no preference between familiar and novel mice or objects.

### *Rng105* ^+/*−*^ mice showed normal learning and memory, but relative difficulty in reversal learning

**Figure 4:**
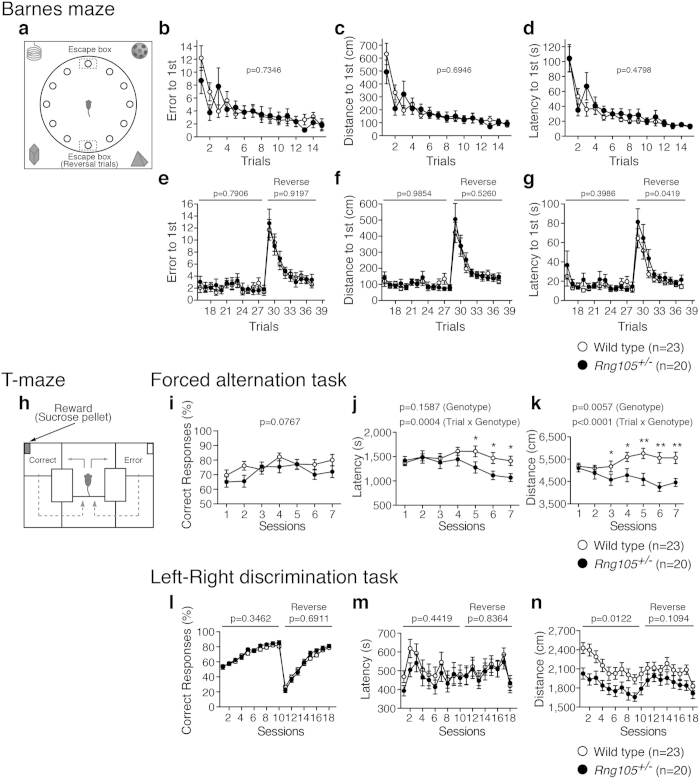
*Rng105*^+/−^ mice performed well on the initial acquisition of memory, but had relative difficulty in reversal learning. (**a**–**g**) Barnes maze test. (**a**) Schematic diagram of the test. Mice are trained to find a target hole connected to an escape box in initial trials. The escape box is moved to the opposite side of the maze in the reversal trials. (**b**–**d**) Initial trials. The number of errors (**b**), total distance traveled (**c**), and latency (**d**), to the first visit to the correct hole in the initial training period (trials 1–15). (**e**–**g**) After a one-month retention interval, the same tests were performed (trials 16–28), followed by the reversal trial (trials 29–37). (**h**–**n**) T-maze test. (**h**) Schematic diagram of the test. In the left-right discrimination task, mice are trained to select a fixed arm with a reward. The rewarded arm is changed to the other side in the reversal sessions. (**i**–**k**) Forced alternation task. Percentage of correct responses to select the arm with the reward (**i**), latency to finish each session (**j**), and total distance traveled (**k**). (**l**–**n**) Left-right discrimination task. Percentage of correct responses to select the arm with the reward (**l**), latency to finish each session (**m**), and total distance traveled (**n**) in the initial training period (sessions 1–10) and reversal training period (sessions 11–18). Data are presented as mean ± S.E.M. P-values for the genotype effect (**b**–**g**,**i**–**n**) and the interaction effect between genotype and session (**j**,**k**) in two-way repeated measures ANOVA are indicated. *p < 0.05, **p < 0.01, one-way ANOVA at the same sessions.

To examine the effect of RNG105 heterozygous knockout on learning and memory, mice were subjected to a Barnes maze test, T-maze test (spontaneous alternation, forced alternation, and left-right discrimination tasks) and contextual and cued fear conditioning tests. Performances were almost the same between wild-type and *Rng105*^+/*−*^ mice in the Barnes maze test (Fig. 4a–d, see Supplementary Fig. S8a–c), T-maze spontaneous alternation test (see Supplementary Fig. S8d–f) and the contextual and cued fear conditioning tests (see Supplementary Fig. S8h–l), which suggested that *Rng105*^+/*−*^ mice had normal reference memory, working memory and contextual memory. However, in the T-maze forced alternation task, *Rng105*^+/*−*^ mice showed less latency and less travel distance to finish the task than wild-type mice as they gained experience, even though the percentage of correct responses to select the rewarded arm was comparable to that of wild-type mice (Fig. 4h–k, see Supplementary Fig. S8g). This tendency was also observed in the T-maze left-right discrimination task: *Rng105*^+/*−*^ mice traveled significantly less distance compared to wild-type mice in the initial training period in which the rewarded arm was fixed in the left or right (sessions 1–10) although the percentage of correct responses was almost the same as wild-type mice (Fig. 4l–n). These results suggested that *Rng105*^+/*−*^ mice showed normal learning and memory, and rather higher performance in specific tasks than wild-type mice.

Furthermore, the mice were subjected to reversal learning in the Barnes maze test and the T-maze left-right discrimination task. In the reversal learning in the Barnes maze test, there were no significant differences in the number of errors or distance to first reach the correct hole between wild-type and *Rng105*^+/*−*^ mice (Fig. 4e,f). However, the *Rng105*^+/*−*^ mice showed longer latency than wild-type mice to first reach the correct hole in the reversal learning (trials 29–37), especially just after the position of the correct hole was changed (Fig. 4g). Next, in the reversal learning in the T-maze left-right discrimination task, correct responses to select the rewarded arm and the latency to finish the task were almost the same between genotypes (Fig. 4l,m). However, the high performance of *Rng105*^+/*−*^ mice, observed in the initial training period, diminished in the reversal training period: although *Rng105*^+/*−*^ mice traveled significantly less distance than wild-type mice to finish the task in the initial training period (sessions 1–10), their travel distance became almost the same as the wild-type mice in the reversal training period (sessions 11–18) (Fig. 4n). These results suggested that *Rng105*^+/*−*^ mice had relative difficulty in reversal learning compared to their normal or high performance in the initial acquisition.

### RNG105 deficiency reduced the cell surface distribution of AMPAR subunit GluR1 in dendrites

The results of behavioral test battery indicated that RNG105 heterozygous knockout led to the reduction in sociality, response to novelty and reversal learning. Previous studies have suggested that there is a link between ASD-like behaviors, including impaired social interaction, and AMPAR down-regulation such as the decreased cell surface distribution of AMPARs^16^,^17^,^18^,^20^,^21^,^22^. Therefore, we examined if RNG105 deficiency also affects the AMPAR cell surface distribution.

**Figure 5:**
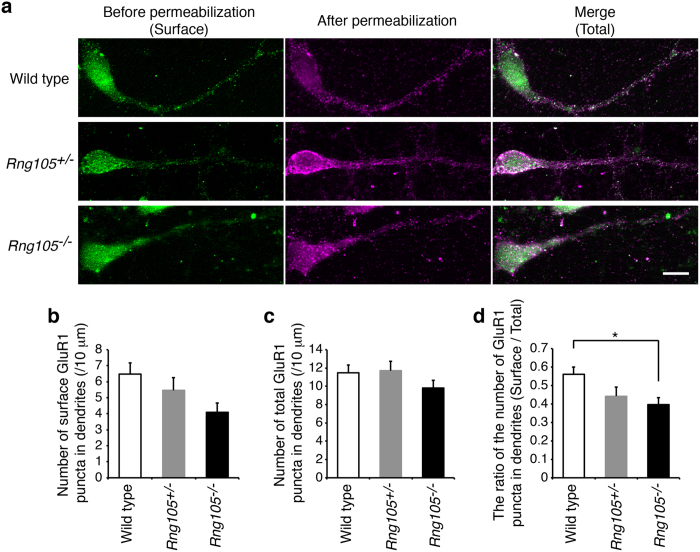
AMPA receptor subunit GluR1 surface distribution was reduced in dendrites of RNG105-deficient neurons. (**a**) Immunostaining for GluR1 in cultured neurons (9 DIV) from cerebral cortexes of E17.5 wild-type, *Rng105*^+/−^ and *Rng105*^−/−^ mice. A cell soma is located on the left side and a primary dendrite elongates toward the right side in each panel. GluR1 staining before permeabilization (green, surface expression), after permeabilization (magenta), and merged images (total expression) are shown. GluR1 is distributed in a punctate manner both in the soma and dendrites. Scale bar, 10 μm. (**b**,**c**) The number of surface (**b**) and total (**c**) GluR1 puncta in dendrites of wild-type, *Rng105*^+/−^ and *Rng105*^−/−^ neurons. (**d**) The ratio of surface/total number of GluR1 puncta in dendrites. Data are presented as means ± S.E.M. Wild type, n = 19; *Rng105*^+/−^, n = 19; *Rng105*^−/−^, n = 21; *p < 0.05, one-way ANOVA followed by Turkey-Kramer test.

First, the surface expression of AMPAR subunit GluR1 was detected by immunostaining of live cultured primary neurons from cerebral cortexes of wild-type and *Rng105*^+/*−*^ mice. (Fig. 5a, left panels). Subsequently, the neurons were fixed, permeabilized and stained with the same antibody to detect intracellular and residual surface GluR1 (Fig. 5a, middle panels). Total GluR1 expression was represented by the sum of the GluR1 images before and after permeabilization (Fig. 5a, right panels). GluR1 was stained in a punctate manner and quantified by counting the puncta number in dendrites. The ratio of surface/total GluR1 puncta number indicated that GluR1 surface distribution in dendrites tended to be less in *Rng105*^+/*−*^ mice than in wild-type mice, though statistically not significant (Fig. 5b–d). Then we performed the same analysis with RNG105 homozygous (*Rng105*^−/−^) mice. *Rng105*^−/−^ neurons showed a larger decrease in dendritic surface GluR1 (Fig. 5a), and quantification revealed that the ratio of surface/total GluR1 puncta number in dendrites was significantly reduced in the *Rng105*^−/−^ neurons (Fig. 5b–d). These results suggested that RNG105 deficiency reduced the cell surface distribution of AMPARs in dendrites. The ratio of surface/total GluR1 appeared to be decreased dose-dependently on RNG105 deficiency, which suggests that even heterozygous knockout of RNG105 impairs the cell surface distribution of AMPARs.

## Discussion

In the present study, we investigated the influences of RNG105 heterozygous knockout on mouse behavior. One of the marked changes in *Rng105*^+/*−*^ mice was sociality. *Rng105*^+/*−*^ mice showed reduced social interaction during an active period in a home cage and reduced social novelty preference in the three-chambered test. The latter change is likely to be characterized as the indistinguishable responses of *Rng105*^+/*−*^ mice to familiarity and novelty, as the *Rng105*^+/*−*^ mice also showed reduced preference for novel objects in the three-chambered test and similar adaptive behaviors between familiar and novel places in the place recognition test. In addition to these changes, *Rng105*^+/*−*^ mice showed relative difficulty in reversal learning compared to initial acquisition in the Barnes maze and T-maze tests. These results suggest that social interaction, responses to novel situations, and flexibility to changes were reduced in *Rng105*^+/*−*^ mice.

*Rng105*^+/*−*^ mice also showed differences from wild-type mice in other tests as follows. In the T-maze forced alternation and left-right discrimination tasks, latency and travel distance to finish the tasks were significantly decreased in *Rng105*^+/*−*^ mice, suggesting that *Rng105*^+/*−*^ mice have different behavioral characteristics and/or better comprehension of the context in specific tasks. In the hot plate test, latency to withdraw the paw was significantly decreased in *Rng105*^+/*−*^ mice, suggesting *Rng105*^+/*−*^ mice were more sensitive to the type of stimulation. *Rng105*^+/*−*^ mice also showed the limb-clasping reflex, which is observed in mice related to several mental disorders^23^,^24^,^25^. Taken together, these behavioral characteristics of *Rng105*^+/*−*^ mice suggested that RNG105 heterozygous knockout could be associated with ASD-like behaviors for the reasons discussed below.

It is well known that ASD is a complex neurodevelopmental disorder^26^,^27^. However, ASD is mainly characterized by three phenotypes: abnormal social interactions, communication deficits and repetitive behaviors^26^,^28^. Previous studies have reported various ASD model mice, e.g., mutations in *Pten*, *Tsc1*, *Tsc2*, *Fmr1*, *Nf1*, *Mecp2*, *Shank1/3*, *Nlgn3/4*, *Nrxn1a*, *Oxt*, *Caps2* and so on, which result in divergent phenotypes^26^,^27^. The ASD model mice do not necessarily show all of the three principal phenotypes, but they do show ASD-like behaviors in multiple tests. The present study demonstrated that *Rng105*^+/*−*^ mice also showed multiple ASD-like behaviors.

The first feature of ASD, i.e., abnormal social interactions, was detected in *Rng105*^+/*−*^ mice. *Rng105*^+/*−*^ mice showed a lack of social novelty preference and reduced social interaction (Fig. 2). However, it is unclear whether the second feature of ASD, i.e., communication deficits, is shown in *Rng105*^+/*−*^ mice. We could not measure the communication ability of *Rng105*^+/*−*^ mice from the sociability tests such as the social interaction test in a home cage, three-chambered social approach test or the social interaction test in a novel environment. It is necessary to measure ultrasonic vocalization and olfaction in order to investigate communication in *Rng105*^+/*−*^ mice. The third feature of ASD, i.e., repetitive behaviors, includes stereotyped behaviors, restricted interests and insistence on sameness^26^,^28^. The stereotypic behaviors of *Rng105*^+/*−*^ mice in the open-field test were not significantly different from wild-type mice, but *Rng105*^+/*−*^ mice had difficulties in the reversal learning tasks (Fig. 4). Reversal learning tasks measure flexible response to changes and are considered to be assays for insistence on sameness^26^,^28^. Although *Rng105*^+/*−*^ mice did not stick to the initial correct hole or arm after the reversal trials in the Barnes maze or T-maze (left-right discrimination task) tests, they had specific impairments in reversal learning, but not in the initial acquisition, suggesting that the phenotypes of *Rng105*^+/*−*^ mice were related to ASD-like behaviors in terms of upset to change.

Other behavioral profiles of ASD are also known, e.g., abnormal sensory function, intellectual disability, increased anxiety, hyper activity, abnormal circadian activity and impairment of motor coordination^27^,^28^. The *Rng105*^+/*−*^ mice were more sensitive to pain in the hot plate test, suggesting that *Rng105*^+/*−*^ mice had abnormal sensory function. However, other features of ASD were not detected in the *Rng105*^+/*−*^ mice.

Furthermore, Asperger’s syndrome is different from typical autism in that Asperger’s patients do not exhibit learning disabilities and, in some cases, exhibit higher cognitive ability. In fact, the Asperger’s patient with a heterozygous nonsense mutation in *Rng105* (*Caprin1*) had an above-average IQ^13^. In this study, *Rng105*^+/*−*^ mice showed normal learning and memory, and even high performances in the T-maze forced alternation and left-right discrimination tasks (Fig. 4), which is reminiscent of the phenotype of Asperger’s patients.

Taken together, RNG105 heterozygous knockout led to ASD-like behaviors as judged from reduction in social interaction, reduced preference for novelty, lower flexibility, tendency to difficulty in reversal learning, and high sensitivity to pain. In particular, *Rng105*^+/*−*^ mice performed well in specific tasks, suggesting that the phenotype of *Rng105*^+/*−*^ mice was related to Asperger syndrome-like behavior.

Well-known ASD risk genes are mainly associated with synaptic functions, including synapse adhesion proteins such as neurexin 1 (*Nrxn1*) and neuroligin 3/4 (*Nlgn3/4*), and scaffold proteins such as Shank 3 (*Shank3*)^26^,^27^,^29^. In addition to synaptic proteins, loss-of-function mutations in translation regulators, such as PTEN phosphatase (*Pten*), Tuberous sclerosis complex 1/2 (*Tsc1/2*) and FMRP (*Fmr1*), lead to ASD^26^,^27^,^29^,^30^. PTEN and TSC1/2 act as negative regulators of the PI3K/mTORC1 signaling pathway which upregulates translation through phosphorylation of eIF4E-binding proteins (4E-BPs) and S6 kinases (S6Ks) in response to excitatory synaptic stimulation in neurons^6^. FMRP is an RNA-binding protein that binds to specific mRNAs and suppresses their translation. The targets of FMRP include mRNAs for synaptic functions and also ASD-linked mRNAs^31^,^6^. Thus, dysregulated translation is suggested to be a risk factor for ASD by affecting synaptic plasticity and function. Considering the already known functions of RNG105^10^,^12^, RNG105 heterozygous knockout could lead to ASD-like behaviors through downregulation of mRNA transport and translation in dendrites. RNG105 is a component of RNA granules in which RNG105 interacts with FMRP^32^, suggesting that altered functions of RNA granules could be associated with ASD.

ASD-associated synaptic proteins and translational regulators typically serve to regulate glutamate receptors including AMPARs^18^. Particularly, in several mutant mice, cell surface distribution of AMPARs is attenuated and the AMPAR-dependent EPSP is reduced^16^,^17^,^20^,^21^,^22^. Similarly to these mutant mice, we showed in the present study that AMPAR surface distribution in dendrites tended to be decreased in *Rng105*^+/*−*^ mice. Although the decrease level was not statistically significant in *Rng105*^+/*−*^ mice, the AMPAR surface reduction reached statistical significance in *Rng105*^−/−^ mice and appeared to be dependent on RNG105 deficiency level, which suggests that even moderate deficiency of RNG105 affects the surface distribution of AMPARs. Although preliminary, we have recently found that, in RNG105 conditional knockout (CaMKIIα-Cre-loxP) mice, localization of mRNAs encoding factors involved in AMPAR surface distribution is significantly reduced in dendritic layers of the hippocampus using RNA-seq and gene ontology analysis (Ohashi, unpublished data). The reduced localization of mRNAs to dendrites could underlie the reduction in the AMPAR surface distribution in dendrites of RNG105-deficient mice. It is a future challenge to investigate if the common feature, downregulation of AMPARs, among ASD-associated mutant mice has a causal link with the neuropathology of ASD.

Translational regulators are reported to be related with learning and memory as well as social behaviors. For example, *Fmr1* knockout mice show abnormal sociality and deficits in learning and memory^33^,^34^,^35^,^36^. Similarly, *Pten* knockout mice, *Tsc1*^+/*−*^ mice and *Tsc2*^+/*−*^ mice also show abnormal social interaction and deficits in spatial memory and contextual memory^37^,^38^,^39^,^40^,^41^. *Rng105*^+/*−*^ mice also showed abnormal social interaction, but their learning and memory were normal. The difference in the learning and memory phenotypes between *Rng105*^+/*−*^ mice and the other translation regulator mutants may be attributed to the difference in their functions, i.e., RNG105 in mRNA transport and the other proteins in translation suppression, or to the modest reduction in the RNG105 protein expression level in *Rng105*^+/*−*^ mice. To investigate whether severe deficiency of RNG105 is associated with learning and memory deficits, a behavioral analysis of RNG105 conditional knockout mice will be required because conventional RNG105 knockout mice were neonatally lethal with respiratory failure^12^. In addition, because some deficits in *Fmr1* knockout mice are rescued by treatment with an mGluR5 inhibitor^42^,^43^ and some ASD-like behaviors in *Pten* knockout mice and *Tsc2*^+/*−*^ mice are rescued by mTOR inhibitors^39^,^40^, treatment of *Rng105*^+/*−*^ mice with these inhibitors will reveal whether RNG105 functions in the mTORC1 signaling and FMRP pathways.

An RNG105 (Caprin1) nonsense mutation was found in the ASD patient^13^, but whether the mutation causes the disorder has been elusive. In the present study, we showed that RNG105 heterozygous knockout in mice led to ASD-like behaviors, which suggests that mutations in the *Rng105* gene can cause ASD-associated phenotypes. The ASD patient has a heterozygous nonsense mutation at Gln-399 of RNG105^13^, which is different from that of the *Rng105*^+/*−*^ mice, which have heterozygous deletion of an exon containing the start codon^12^, suggesting that *Rng105*^+/*−*^ mice do not strictly mimic the patient. However, the mutant RNG105 in the ASD patient may not have a dominant effect because abnormal mRNAs containing premature nonsense codons are rapidly degraded by nonsense-mediated mRNA decay (NMD)^44^,^45^, and even if a partial-length RNG105 protein (amino acid 1-398) is synthesized, the protein lacks both the RGG box (amino acid 606-634) and RG-rich (amino acid 636-690) domains required for RNA binding and RNA granule localization^10^. Thus, the ASD phenotypes of the patient are supposed to be caused by the RNG105 deficiency, which may be consistent with the results in which *Rng105*^+/*−*^ mice showed ASD-like behaviors. Of course, this does not exclude the possibility that gain-of function mutations in RNG105 lead to ASD-associated phenotypes. Further identification of mutations in the *Rng105* gene in various ASD patients and analyses of such mutant RNG105 in mice and *in vitro* will confirm the hypothesis that RNG105 is a risk gene for ASD including Asperger’s syndrome.

## Methods

### Ethics statement

All animal care, experiments and behavioral testing procedures were approved by the Institutional Animal Care and Use Committee of the National Institutes of Natural Sciences, and performed in accordance with the guidelines from the National Institutes of Natural Sciences and the Science Council of Japan.

### RNA isolation and quantitative reverse transcription (RT)-PCR

Total RNA was extracted from adult mouse tissues using ISOGEN (Nippon Gene, Tokyo, Japan) following the manufacturer’s protocol. RT was performed with M-MLV-Reverse Transcriptase (Invitrogen, Carlsbad, CA) and quantitative PCR was performed with SYBR Premix Ex Taq II (Tli RNaseH Plus) (TaKaRa, Shiga, Japan) on an Applied Biosystems 7500 real-time PCR system (Applied Biosystems, Carlsbad, CA) following the manufacturer’s protocols. The primer sequences of *Rng105* were 5′-GGAGCAAGTGGATGAGTGGA-3′ and 5′-GTCAAAGAGTGGGGCTCTGG-3′.

### Western blotting

Extracts of adult mouse tissues were prepared from wild-type and *Rng105*^+/*−*^ mice by homogenization and sonication in a Laemmli sample buffer. After boiling for 5 min, the extracts were loaded onto SDS-polyacrylamide gels and transferred to polyvinylidene difluoride membranes. The membranes were probed with an anti-RNG105 polyclonal antibody^10^, an anti- Ras-GAP SH3 domain binding protein (G3BP) antibody^10^, an anti-α-tubulin antibody (PM054, Medical & Biological Laboratories, Nagoya, Japan), and an anti-GluR1 antibody (PC246, Merck Millipore, Billerica, MA). Biotinylated secondary antibody (GE Healthcare, Buckinghamshire, UK) and alkaline phosphatase-conjugated streptavidin (GE Healthcare) were used for detection with a bromochloroindolyl phosphate/nitro blue tetrazolium solution.

In quantification experiments for RNG105, the tissue extracts (cerebrum, cerebellum, spinal nerve and liver, 40 μg per lane; thymus, 20 μg per lane) and a standard dilution series of each tissue extract from wild-type mice (cerebrum, cerebellum, spinal nerve and liver, 10, 20, 30, 40, 50 μg per lane; thymus, 5, 10, 15, 20, 25 μg per lane) were loaded on the same gel. RNG105 protein expression level was quantified by measuring the band intensity on the membrane using Adobe Photoshop software. A standard curve was generated from the standard dilution series and used to calculate the relative expression level of RNG105 in each tissue from wild-type and *Rng105*^+/*−*^ mice. Quantification experiments for α-tubulin were conducted in the same way. The amount of extracts loaded on the gel was different from the experiments for RNG105 as indicated in Supplementary Fig. S1.

### Neuronal culture

Dissociated cerebral cortical neurons were prepared from embryonic day 17.5 (E17.5) individual littermates. Neurons were plated at a density of 6.4 × 10^4^ cells/cm^2^ onto poly-D-lysine-coated coverslips of glass bottom culture dishes (MatTek, Ashland, MA) in Neurobasal-A medium (Thermo Fisher Scientific, Waltham, MA) containing B-27 supplement (Thermo Fisher Scientific), 0.5 mM glutamine and 25% Neuron culture medium (Sumitomo Bakelite, Tokyo, Japan). Cultures were maintained at 37 °C in a 5% CO_2_ incubator.

### Immunocytochemistry

Cultured neurons were used at 9 d *in vitro* (DIV) for GluR1 immunostaining as described previously with modifications^46^,^47^. Live neurons were incubated with the anti-GluR1 antibody (1:15; PC246, Merck Millipore) for 1 h at 37 °C in a 5% CO_2_ incubator. After the cells were washed three times in Neurobasal-A medium, they were fixed for 20 min at room temperature with 3.7% paraformaldehyde in PBS. Then the fixed neurons were blocked for 30 min in 10% fetal calf serum (FCS) in DMEM, and incubated with Alexa Fluor 488-conjugated anti-rabbit IgG secondary antibody (Thermo Fisher Scientific) in 10% FCS in DMEM for 3 h at room temperature without permeabilization to label cell surface GluR1. After the cells were washed in PBS, they were fixed again and permeabilized for 10 min with 0.25% TritonX-100 in PBS. After the cells were blocked, they were incubated with the anti-GluR1 antibody (1:50) for 12 h at 4 °C and then with Cy3-conjugated anti-rabbit IgG secondary antibody (Jackson ImmunoResearch, West Grove, PA) for 3 h at room temperature to label intracellular and residual surface GluR1.

### Image analysis

Neurons were imaged using an Olympus IX83 inverted fluorescence microscope (Olympus, Tokyo, Japan) with a 40× objective lens and an ORCA-R2 digital CCD camera (Hamamatsu Photonics, Hamamatsu, Japan). Dendritic regions in the images were selected and converted into binary images using the MaxEntropy threshold algorithm in NIH ImageJ software. The number of GluR1 puncta in dendrites was counted using magic wand tool and analysis tool in Adobe Photoshop software. The length of dendrites was measured and the number of GluR1 puncta per 10 μm of dendrite was calculated. To count the number of total GluR1 puncta, the binary images of before and after permeabilization conditions were merged and used.

### Animals and design of behavioral experiments

*Rng105*^+/*−*^ mice were generated previously^12^, and backcrossed for more than 10 generations on the C57BL/6 background. *Rng105*^+/*−*^ mice and wild-type littermates were prepared by crossing wild-type female mice and *Rng105*^+/*−*^ male mice. All behavioral tests were carried out with male mice. Mice were group-housed (4 mice per cage, two pairs of wild-type and *Rng105*^+/*−*^ mice) in a room with a 12-hour light/dark cycle. The order in which mice were subjected to tests was counterbalanced. The order of behavioral tests is listed in Table 1. Raw data were disclosed in the Mouse Phenotype Database (http://www.mouse-phenotype.org/).

### Three-chambered social approach test

The test for sociability and social novelty preference was conducted as previously described^48^,^15^. For habituation to the test environment, stranger mice were put into a small cylindrical cage with vertical bars and placed in a corner of a chamber prior to the test. Test mice were placed in the middle chamber and allowed to explore and habituate to the chambers for 10 min just before the first session. In the first session, an unfamiliar mouse (stranger 1) was put into the cage and placed in a corner of the left or right side chamber. The selection of left or right side chamber was counterbalanced across test mice. An empty cage was placed in a corner of the other side chamber. The test mouse was then placed in the middle chamber and allowed to explore the three chambers for 10 min. In the second session, another unfamiliar mouse (stranger 2) was placed in the cage that had been empty during the first session. The test mouse was then placed in the middle chamber and allowed to explore for 10 min. Data acquisition and analysis were performed automatically using ImageCSI (see Section, “Data analysis in behavioral tests”). Heat maps for the average traces were generated by merging the traces of the same genotype mice using Image J software.

### Social interaction test in a home cage

A social interaction test in a home cage was conducted as previously described^15^. Two mice of the same genotype, which had been housed separately, were placed together in a home cage. Their social interaction and locomotor activity were monitored for seven days. Social interaction was measured by counting the number of particles detected in each frame, i.e., two particles indicated that the two mice were separate from each other, and one particle indicated that they were in contact with each other. Data acquisition and analysis were performed automatically using ImageHCSI (see Section, “Data analysis in behavioral tests”).

### Social interaction test in a novel environment

Two mice were placed in a box and allowed to explore freely for 10 min. They were identical genotypes and previously housed in different cages. Data acquisition and analysis were performed automatically using ImageSI (see Section, “Data analysis in behavioral tests”).

### Novel object recognition test

The method was the same as the three-chambered social approach test except for using objects instead of stranger mice. Objects of four types, a black sphere, a black cone, a column with vertical stripes and a cube with black dots, were used. The cylindrical cage was not used in this test.

### Place recognition test

The place recognition test was conducted as previously described^49^. The square-type (S) chamber (30 × 30 × 30 cm) made of transparent plastic with steel grids on the floor and the circle-type (C) chamber (30 cm height, 34 cm diameter) made of white plastic were used. During the learning session, the mice were placed in chamber S or chamber C, and allowed to freely explore for 6 min. 24 hours after the learning session, the mice were placed in the experienced chamber or the novel chamber, and allowed to explore for 3 min. Data acquisition and analysis were performed automatically using ImageOF (see Section, “Data analysis in behavioral tests”).

### Barnes maze test

The Barnes maze test was conducted as previously described^15^. An escape box, which contained paper cage bedding, was located under the target hole. Mice were trained for 15 trials. 24 hours after the last training, a probe trial (PT1) was performed without the escape box. 1 month after the PT1, another probe trial (PT2) was performed to evaluate memory retention. After PT2, mice were trained for an additional 13 trials, in which the location of the target hole was not changed from the initial training trials. After that, mice were subjected to a reversal training session. In the reversal training, the location of the target hole was changed to the opposite side of the maze, and the mice were trained for 9 trials. 24 hours after the last reversal training, a probe trail (PT3) was performed. Data acquisition and analysis were performed automatically using ImageBM (see Section, “Data analysis in behavioral tests”).

### T-maze

The T-maze tests were conducted as previously described^50^ using the automatic modified T-maze apparatus (O’Hara & Co., Tokyo, Japan). Data acquisition and analysis were performed automatically using ImageTM (see Section, “Data analysis in behavioral tests”).

In the spontaneous alternation task, mice were allowed to freely run for 10 laps in a session, and subjected to 5 sessions in total. A correct response in each lap indicated that mice chose the opposite direction from the last lap.

Prior to the forced alternation and left-right discrimination tasks, mice went on a restricted diet. Their body weights were reduced to about 80% of that before the dietary restriction and maintained throughout the experiments. In the forced alternation task, each session consisted of a forced-choice run followed by a free-choice run. In the forced-choice run, a sucrose pellet was delivered to the food tray in one of the left or right arms with only the door leading to the rewarded arm opened. In the free-choice run, a sucrose pellet was delivered to the opposite tray to that in the forced-choice run, and both the doors leading to the left and right arms were opened. Mice were subjected to 10 consecutive sets of forced- and free-choice runs in a session, and subjected to 7 sessions in total. After that, mice were subjected to the delayed alternation task. In the task, 3-, 10-, 30-, or 60-sec delays were inserted between the forced- and free-choice runs. Mice were subjected to 3 sessions.

In the left-right discrimination task, each session consisted of free-choice runs. A sucrose pellet was always delivered to the same food tray in one of the arms. The location of the rewarded arm was fixed for a mouse but counterbalanced across mice. Mice were subjected to 10 consecutive laps in a session, and subjected to 10 sessions in total. After that, mice were subjected to reversal learning sessions (10 laps per session, 8 sessions in total). In the reversal learning sessions, the arm with the sucrose pellet was changed to the opposite side to that in the initial sessions.

### Data analysis in behavioral tests

Behavioral data were obtained automatically by applications (ImageSI, ImageCSI, ImageHCSI, ImageOF, ImageBM and ImageTM) based on the public domain NIH Image program and ImageJ program, and modified for each test by Tsuyoshi Miyakawa (O’Hara & Co.). Statistical analysis was conducted using StatView (SAS Institute, Cary, NC). Data were analyzed by Student’s t-test, paired t-test, one-way ANOVA, or two-way repeated measures ANOVA. Values in graphs are presented as mean ± S.E.M.

## Additional Information

**How to cite this article**: Ohashi, R. *et al.* Comprehensive behavioral analysis of RNG105 (Caprin1) heterozygous mice: Reduced social interaction and attenuated response to novelty. *Sci. Rep.* **6**, 20775; doi: 10.1038/srep20775 (2016).

## Supplementary Material

Supplementary Information

## Figures and Tables

**Table 1:** Comprehensive behavioral test battery of *Rng105* ^+/−^ mice.

Test	Age (weeks old)	Phenotypes in *Rng105*^+/−^mice	Results
Body weight, body temperature	12	NS	Sup. 2
Wire hang	12	NS	Sup. 2
Grip strength	12	NS	Sup. 2
Light/dark transition	12	NS	Sup. 6
Open field	13	NS	Sup. 3
Elevated plus maze	14	NS	Sup. 6
Hot plate	14	Reduced latency to withdraw their paws	Sup. 5
Social interaction test in a novel environment	14	NS	Fig. 2
Rotarod	15	NS	Sup. 4
Three-chambered social approach	17	Reduced preference for social novelty	Fig. 2
Startle response/prepulse inhibition	18	NS	Sup. 5
Porsolt forced swim	18	Reduced immobility	Sup. 7
Gait analysis	19	Reduced stride length	Sup. 4
Barnes maze	21–28	Increased latency to reach the correct hole in reversal learning	Fig. 4
			Sup. 8
T-maze (spontaneous alternation)	33–34	NS	Sup. 8
Tail suspension	35	NS	Sup. 7
Beam test	38	NS	Sup. 4
Limb-clasping reflexes	40	Increased limb-clasping reflexes	Sup. 4
Novel object recognition	40	Reduced preference for object novelty	Fig. 3
T-maze (forced alternation)	44–51	Reduced latency and distance to finish the task	Fig. 4
			Sup. 8
T-maze (left-right discrimination)	51–53	Reduced distance to finish the task in initial learning but not in reversal learning	Fig. 4
Contextual and cued fear conditioning	56–60	NS	Sup. 8
Social interaction test in a home cage	60–61	Reduced social interaction during active period	Fig. 2
Marble burying test	63	NS	Sup. 6
Place recognition test	70	Reduced motility in a novel chamber	Fig. 3

NS: no significant differences.
