# Supplementary material for: Comprehensive behavioral analysis of RNG105 (Caprin1) heterozygous mice: Reduced social interaction and attenuated response to novelty
Source: Sci Rep. 2016 Feb 11;6:20775. doi: 10.1038/srep20775 (PMC4749962; doi:10.1038/srep20775)
Supplement: Supplementary Information [file srep20775-s1.pdf]

# Supplementary information

# Comprehensive behavioral analysis of RNG105 (Caprin1) heterozygous mice: Reduced social interaction and attenuated response to novelty

Rie Ohashi[1,2], Keizo Takao[4,5,6], Tsuyoshi Miyakawa[4,7] & Nobuyuki Shiina[1,2,3]

[1]Laboratory of Neuronal Cell Biology, National Institute for Basic Biology, Okazaki, Aichi, Japan, [2]Department of Basic Biology, SOKENDAI, Okazaki, Aichi, Japan, [3]Okazaki Institute for Integrative Bioscience, Okazaki, Aichi, Japan, [4]Section of Behavior Patterns, National Institute for Physical Science, Okazaki, Aichi, Japan, [5]Department of Physiology, SOKENDAI, Okazaki, Aichi, Japan, [6]Division of Animal Resources and Development, Life Science Research Center, University of Toyama, Toyama, Japan, and [7]Division of Systems Medical Science, Institute for Comprehensive Medical Science, Fujita Health University, Toyoake, Aichi, Japan.

Correspondence and requests for materials should be addressed to Nobuyuki Shiina (nshiina@nibb.ac.jp)

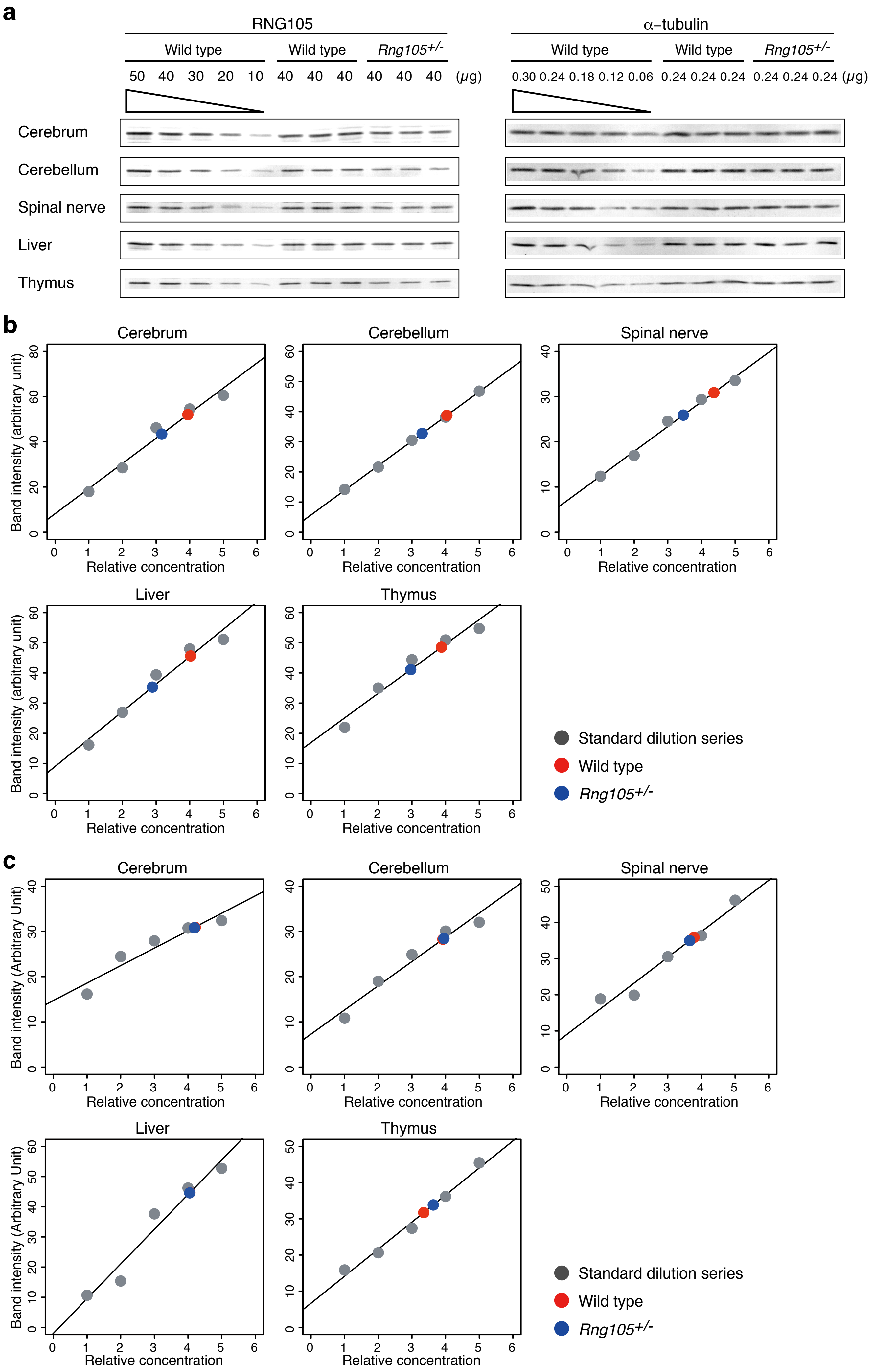
a
RNG105
α–tubulin
Wild type
Wild type
Rng105+/-
50 40 30 20 10 40 40 40 40 40 40 (μg)
0.30 0.24 0.18 0.12 0.06 0.24 0.24 0.24 0.24 0.24 0.24 (μg)
Cerebrum
Cerebellum
Spinal nerve
Liver
Thymus
b
c
Band intensity (arbitrary unit)
Band intensity (Arbitrary Unit)
Relative concentration
Standard dilution series
Wild type
Rng105+/-

**Supplementary Figure S1 | Quantification of RNG105 and α-tubulin protein expression in *Rng105*$^{+/-}$ mice.**

(**a**) Western blotting of extracts from adult mouse cerebrum, cerebellum, spinal nerve, liver and thymus with an anti-RNG105 antibody and an anti-α-tubulin antibody. Lanes 1-5, dilution series of extracts from wild-type mouse tissues; lanes 6-8, extracts from wild-type mouse tissues; lanes 9-11, extracts from *Rng105*$^{+/-}$ mouse tissues. Numbers indicate the amount of extracts (μg) loaded on each lane. The amount of thymus extracts loaded on each lane was 1/2 of the indicated number for RNG105. The amount of liver and thymus extracts loaded on each lane was ×32 and ×8 of the indicated number, respectively, for α-tubulin. (**b, c**) Representative quantitative analysis of RNG105 protein (b) and α-tubulin protein (c) in each tissue. Standard curves were obtained from the band density of RNG105 and α-tubulin in the dilution series (gray dots). RNG105 and α-tubulin expressions were quantified by plotting the densitometric values of the RNG105 and α-tubulin bands in wild-type extracts (red dots) and *Rng105*$^{+/-}$ extracts (blue dots) on the standard curves.

General health

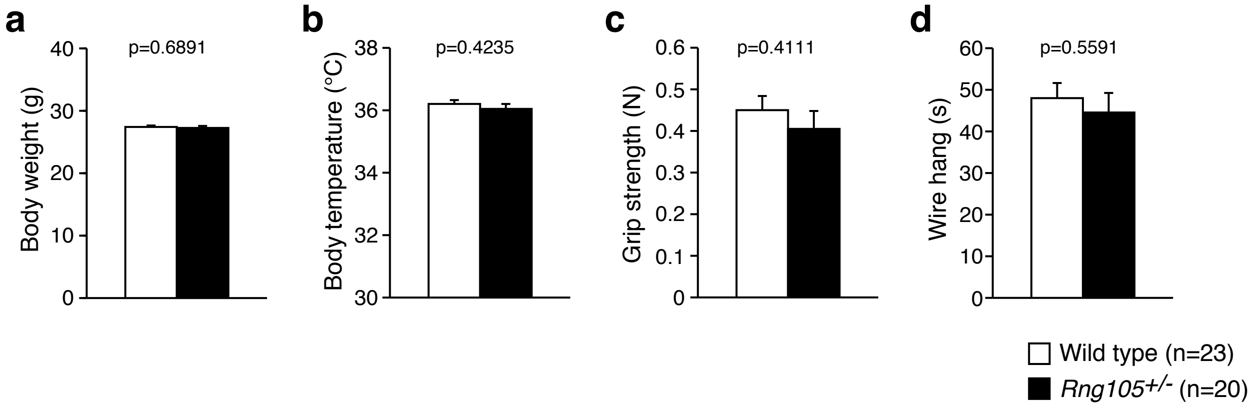


**Supplementary Figure S2 | Physical characteristics and muscular strength were normal in *Rng105*$^{+/-}$ mice.**

(**a**) Body weight. (**b**) Body temperature. (**c**) Grip strength. (**d**) Latency to fall in the wire hang test. Data are presented as mean ± standard error of the mean (S.E.M). P-values from one-way ANOVA are indicated.

Open field test

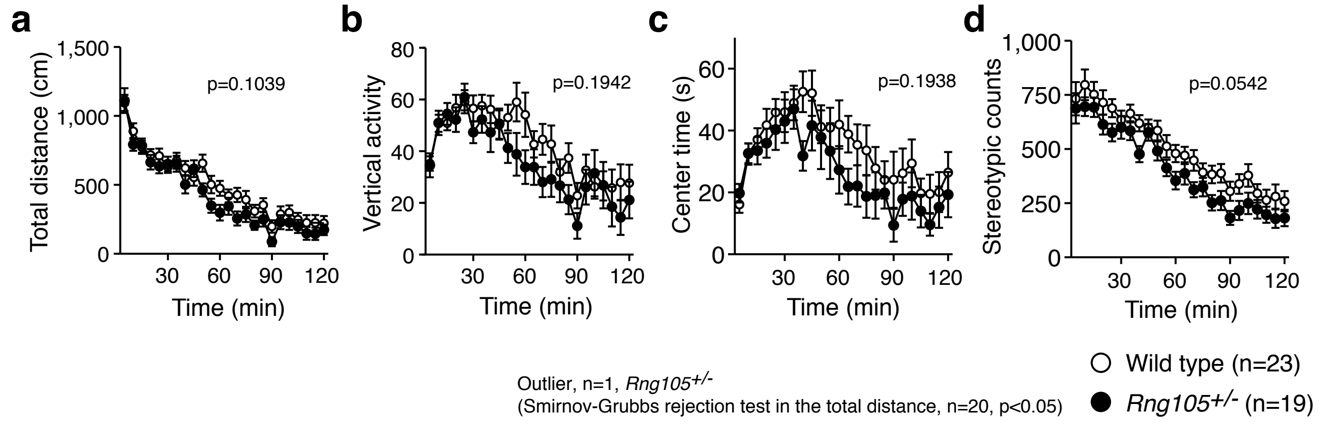


**Supplementary Figure S3 | There were no significant differences in the open field test between wild-type and *Rng105*$^{+/-}$ mice.**

(**a**) Total distance traveled. (**b**) Counts of vertical activity. (**c**) Time spent in the center area. (**d**) Counts of stereotypic behavior. Data are presented as mean ± S.E.M. P-values for the genotype effect in two-way repeated measures ANOVA are indicated.

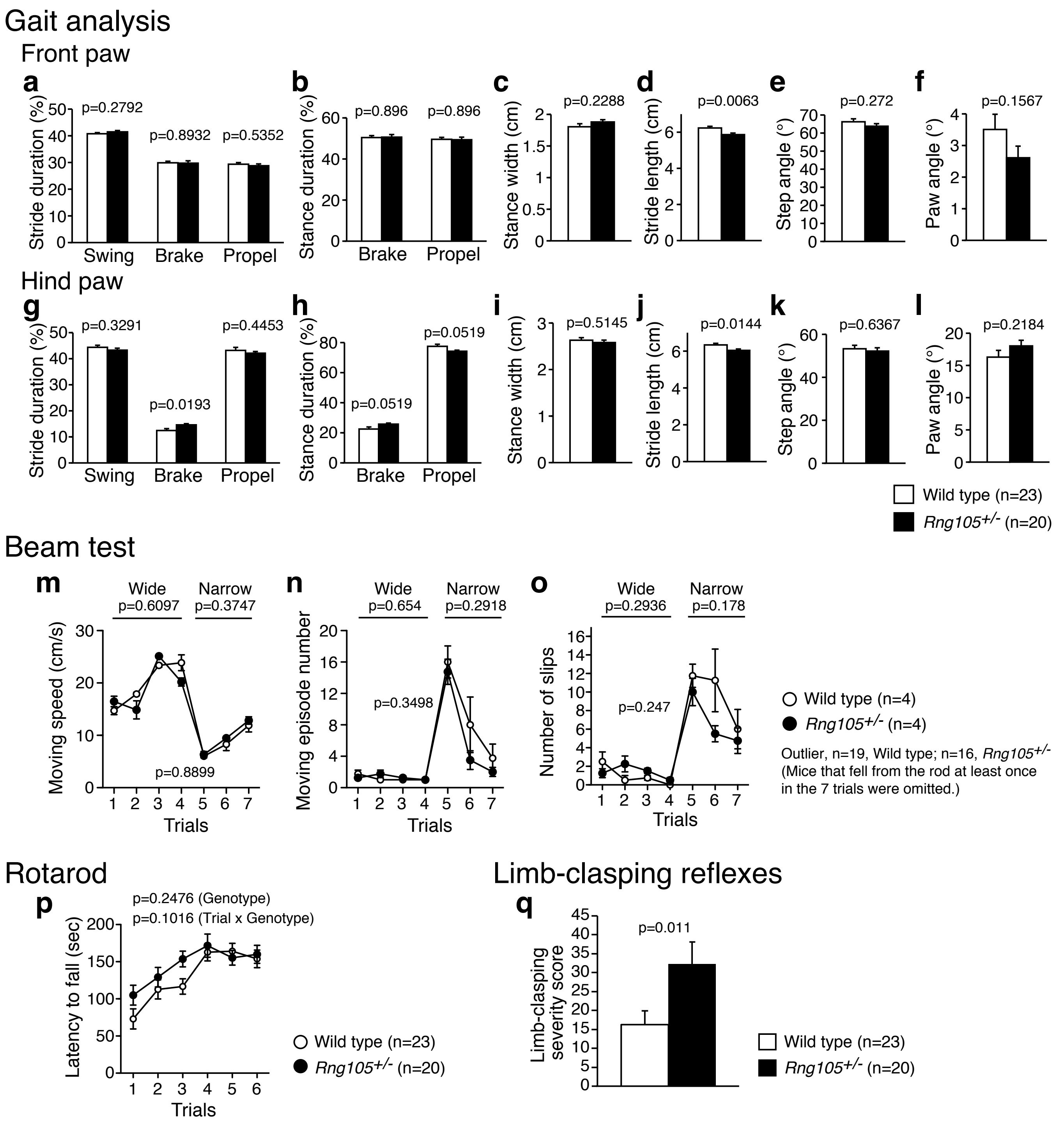


**Supplementary Figure S4 | Normal motor skills and motor learning except for reduced stride length and increased limb-clasping reflexes in *Rng105*$^{+/-}$ mice.**

(**a-l**) Gait analysis of front paw (a-f) and hind paw (g-l). Percentage of the duration of each gait index in a stride (a and g), percentage of the duration of each gait index in a stance (b and h), stance width (c and i), stride length (d and j), step angle (e and k), and paw angle (f and l). (**m-o**) Beam test. Moving speed (m), the number of transitions from stop to go on the rod (n), the number of slips on the rod (o). A wide rod was used in trials 1-4, and a narrow rod was used in trials 5-7. Mice that fell from the rod at least once in the seven trials were omitted from the data analysis. (**p**) Latency to fall from the rod in the rotarod test. (**q**) Limb-clasping reflexes. Data are presented as mean ± S.E.M. P-values from one-way ANOVA (a-l), student's t-test (q), and p-values for the genotype effect (m-p) and the interaction effect between genotype and trial (p) in two-way repeated measures ANOVA are indicated.

## Hot plate test

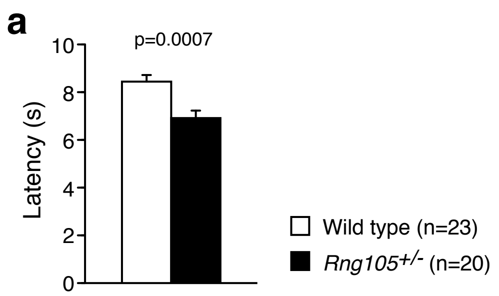


## Startle response/prepulse inhibition

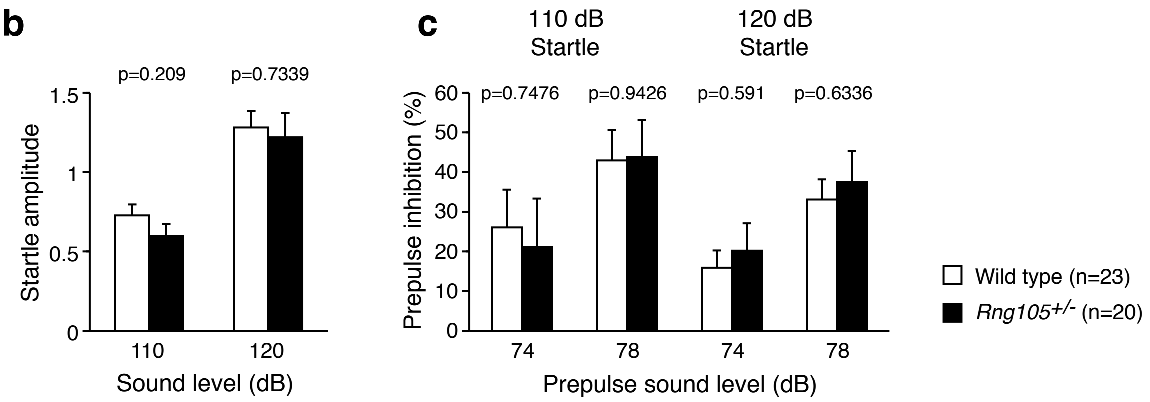


**Supplementary Figure S5 | *Rng105*$^{+/-}$ mice showed a rapid response in the hot plate test, but normal startle response in the prepulse inhibition test.**

(**a**) Hot plate test. Latency to withdraw the paw from the hot plate. (**b** and **c**) Startle response/prepulse inhibition test. (**b**) Acoustic startle response to single 110 dB and 120 dB stimuli. (**c**) Prepulse inhibition of the acoustic startle response with 74 dB and 78 dB prepulse sounds. Data are presented as mean ± S.E.M. P-values from one-way ANOVA are indicated.

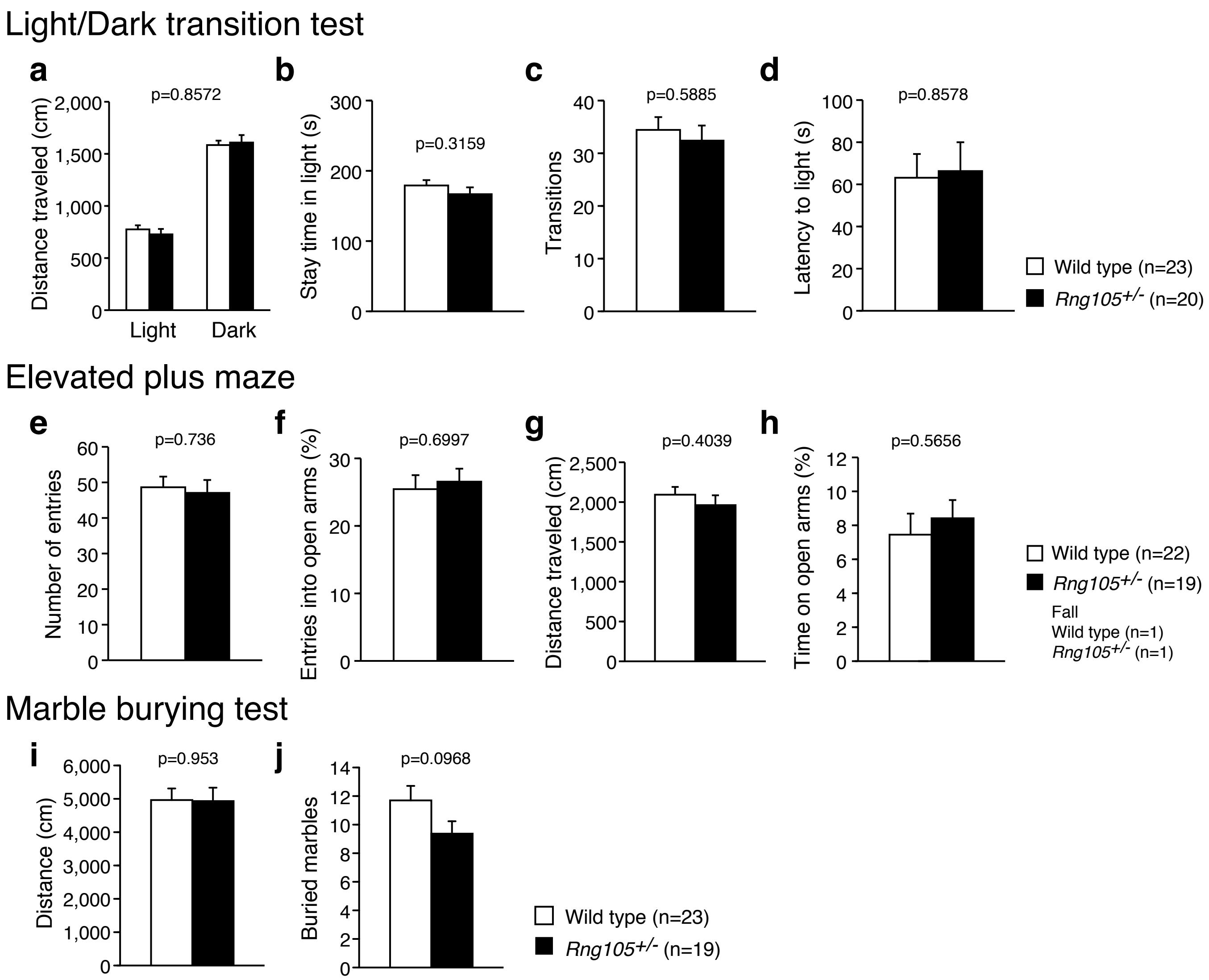


**Supplementary Figure S6 | Anxiety-like behaviors and obsessive-compulsive disorder (OCD)-like behavior were normal in *Rng105*$^{+/-}$ mice.**

(**a**-**d**) Light/dark transition test. Total distance traveled (a), stay time in the light compartment (b), the number of light/dark transitions (c), and latency to the light compartment (d). (**e**-**h**) Elevated plus maze test. The number of arm entries (e), percentage of entries into open arms (f), total distance traveled (g), and percentage of time spent on open arms (h). (**i** and **j**) Marble burying test. Total distance traveled (i), the number of buried marbles (j). Data are presented as mean ± S.E.M. P-values for the genotype effect in two-way repeated measures ANOVA (a) and p-values from one-way ANOVA (b-j) are indicated.

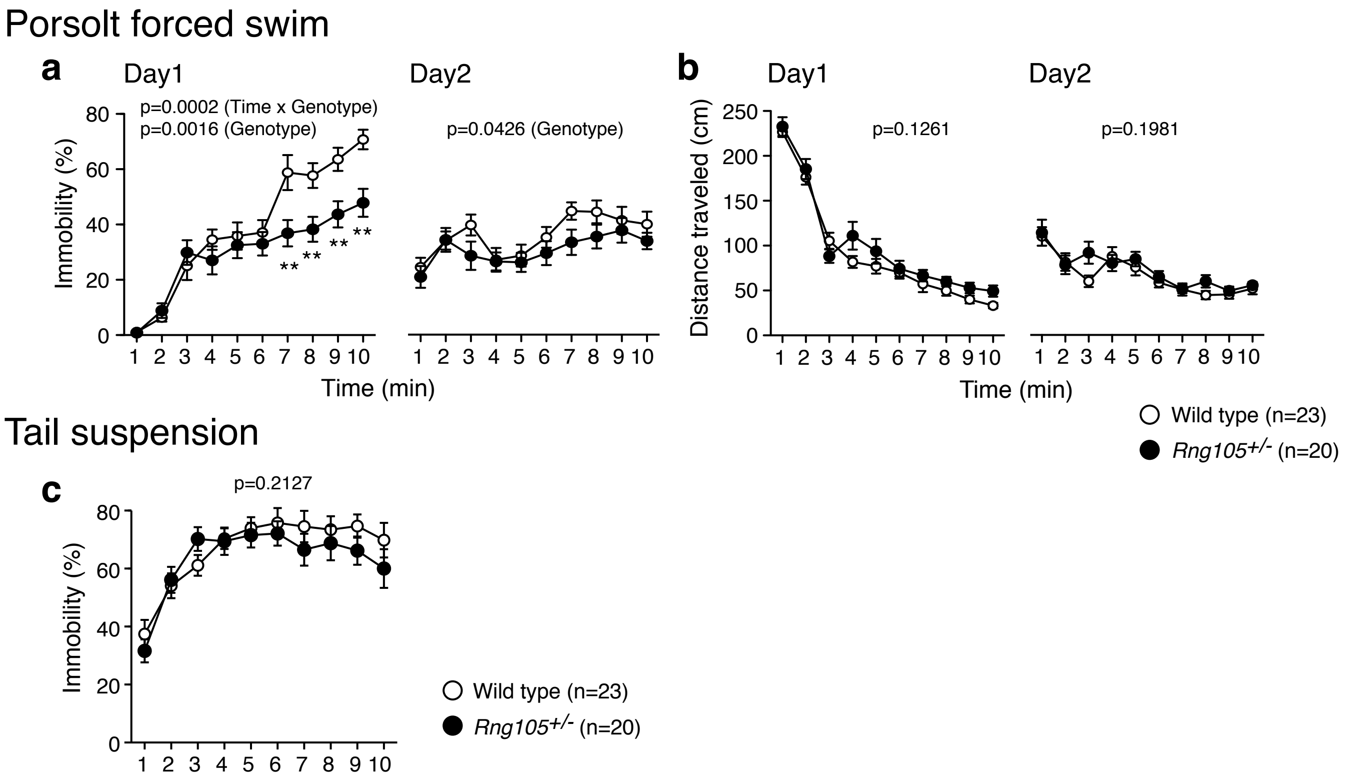


**Supplementary Figure S7 | Depressive-like behaviors: reduced immobility in the Porsolt forced swim test but not in the tail suspension test in *Rng105*$^{+/-}$ mice.**

(**a** and **b**) Porsolt forced swim test. Percentage of immobility time on day 1 and day 2 (a), and distance traveled on day 1 and day 2 (b). (**c**) Percentage of immobility time in the tail suspension test. Data are presented as mean ± S.E.M. P-values for the genotype effect (a-c) and the interaction effect between genotype and time (a) in two-way repeated measures ANOVA are indicated. **p<0.01, one-way ANOVA at the same time points.

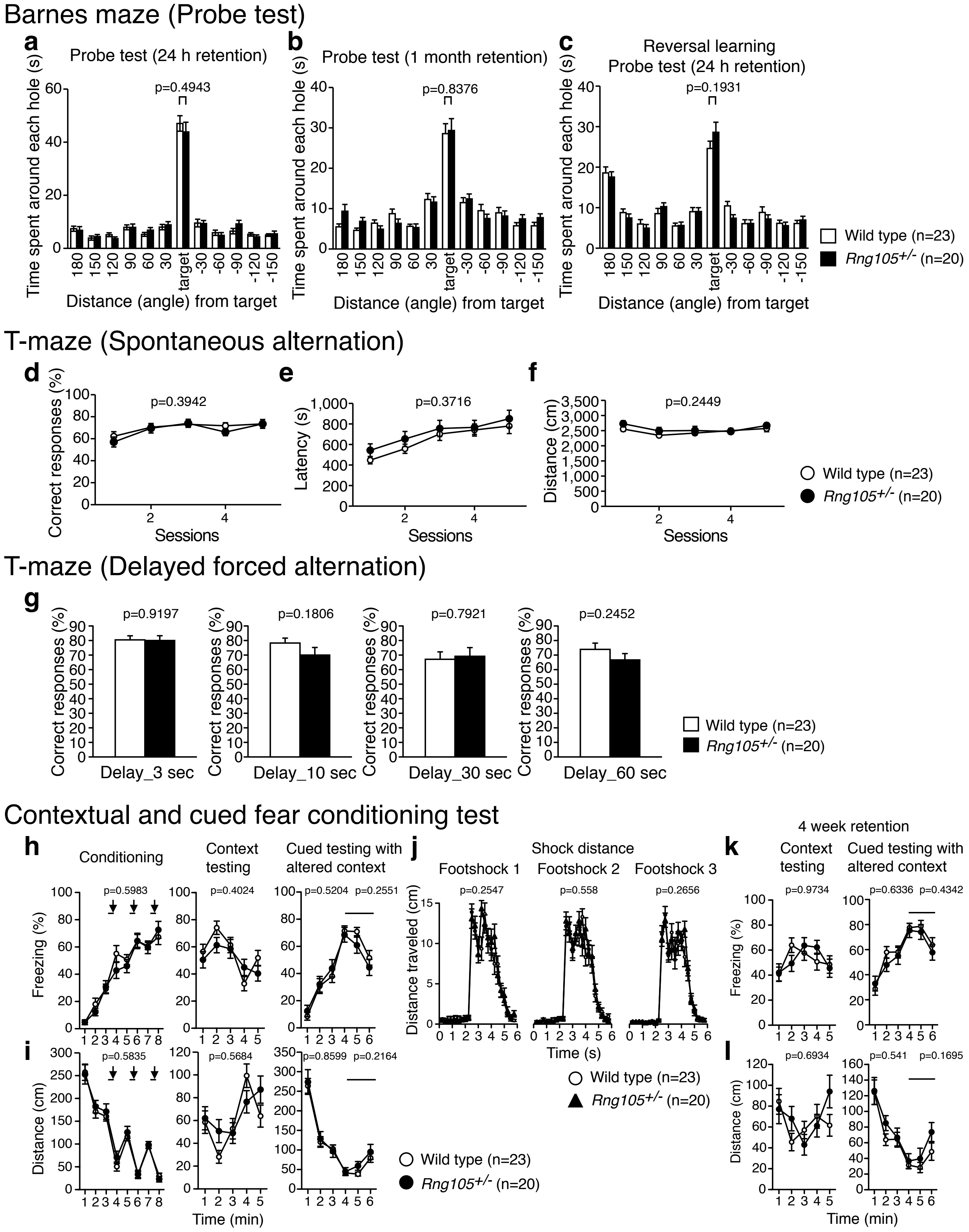
Barnes maze (Probe test)
a
Probe test (24 h retention)
p=0.4943
b
Probe test (1 month retention)
p=0.8376
c
Reversal learning
Probe test (24 h retention)
p=0.1931
Time spent around each hole (s)
Distance (angle) from target
180
150
120
90
60
30
target
-30
-60
-90
-120
-150
Wild type (n=23)
Rng105+/- (n=20)
T-maze (Spontaneous alternation)
d
p=0.3942
Correct responses (%)
e
p=0.3716
Latency (s)
f
p=0.2449
Distance (cm)
Sessions
Wild type (n=23)
Rng105+/- (n=20)
T-maze (Delayed forced alternation)
g
p=0.9197
p=0.1806
p=0.7921
p=0.2452
Correct responses (%)
Delay_3 sec
Delay_10 sec
Delay_30 sec
Delay_60 sec
Wild type (n=23)
Rng105+/- (n=20)
Contextual and cued fear conditioning test
h
Conditioning
p=0.5983
Context testing
p=0.4024
Cued testing with altered context
p=0.5204
p=0.2551
Freezing (%)
i
p=0.5835
p=0.5684
p=0.8599
p=0.2164
Distance (cm)
Time (min)
Wild type (n=23)
Rng105+/- (n=20)
j
Shock distance
Footshock 1
Footshock 2
Footshock 3
p=0.2547
p=0.558
p=0.2656
Distance traveled (cm)
Time (s)
Wild type (n=23)
Rng105+/- (n=20)
4 week retention
k
Context testing
p=0.9734
Cued testing with altered context
p=0.6336
p=0.4342
Freezing (%)
l
p=0.6934
p=0.541
p=0.1695
Distance (cm)
Time (min)

**Supplementary Figure S8 | Learning and memory were normal in *Rng105*$^{+/-}$ mice.**

(**a**-**c**) Probe tests in the Barnes maze test. Time spent around each hole in probe tests performed at 24 h (a) and one month (b) after the initial trial 15 in Fig. 4, and at 24 h after the last reversal trial 37 in Fig. 4 (c). (**d**-**f**) T-maze spontaneous alternation task. Percentage of alternation (d), latency to finish each trial (e), and total distance traveled (f). (**g**) T-maze delayed forced alternation task. Delay time of 3, 10, 30 or 60 sec was inserted between the forced-choice run and the free-choice run. The percentage of correct responses to select the rewarded arm in the delayed trials is shown. (**h**-**l**) Contextual and cued fear conditioning test. (**h** and **i**) Percentage of freezing time (h) and distance traveled (i) in conditioning trials (left panels), in context test (middle panels) and in cued test (right panels). The tests were conducted at 24 h after the conditioning. (**j**) Distance traveled just after receiving 2-sec footshock. (**k** and **l**) After a 4-week retention interval, the same tests were conducted as in h and i. Bars indicate tone presentation (h, i, k and l) and arrows indicate the presentation of footshock (h and i). Data are presented as mean ± S.E.M. P-values from one-way ANOVA (a-c and g) and p-values for the genotype effect in two-way repeated measures ANOVA (d-f, h-l) are indicated.

## Supplementary Methods

### Body weight, body temperature and neuromuscular examination

Mice were placed in a stainless bowl and body weight was measured using a scale. The rectal temperature was measured as body temperature. Neuromuscular strength was examined by grip strength and wire-hang tests. A grip strength meter (O'Hara & Co., Tokyo, Japan) was used to measure the mouse forelimb grip strength. Mice were held by the tail to allow their forelimbs to grip the wire grid. The mice were then gently pulled backward by the tail until they released the wire grid. The maximum force generated by the mouse forelimbs was recorded. Each mouse was tested three times and the greatest value was used for the analysis. In the wire-hang test, a box (22 × 22 × 30 cm) with a wire mesh grid (10 × 10 cm) on its upper surface (O'Hara & Co.) was used. Mice were placed on the wire mesh grid, and it was inverted gently, so that the mice gripped the wire. Latency to fall from the wire was recorded with a 60 sec cutoff time.

### Open field test

An open field apparatus (40 × 40 × 30 cm; AccuScan Instruments, Columbus, OH) was illuminated at 100 lux. Total distance traveled, vertical activity (rearing measured by counting the number of photobeam interruptions), time spent in the center area of the chamber, and the stereotypic counts were recorded for 120 min using the VeraMax system (AccuScan Instruments).

### Gait analysis

The treadmill gait analysis was performed using the DigiGait imaging system (Mouse Specifics Inc., Quincy, MA)[51,52]. Mice were placed on a transparent treadmill belt moving at a speed of 24 cm/sec. Digital images of paw placement were recorded through the transparent treadmill belt. The images for 3 sec (150 frame/sec) were analyzed using the software DigiGait 11.1 (Mouse Specifics Inc). DigiGait automatically identified the stance and swing components of the stride and calculated the stance width, stride length, step angle and paw angle.

### Beam test

A wide rod (100 cm long, 2.5 cm diameter) and a narrow rod (100 cm long, 1 cm diameter) were used. The rod was placed 35 cm above the floor and a black box (20 × 20 × 20 cm) used as a goal was placed at the end of the rod. Mice were placed at the start end, and walked to the goal box. The mice were subjected to 4 trials using the wide rod, and 3 trials using the narrow rod. The moving speed, number of slips on the rod and number of transitions from stop to go on the rod were measured.

**Rotarod test**

An accelerating rotarod (UGO Basile, Varese, Italy) was used. Mice were placed on the rotation rod (3 cm diameter) and the latency of the mice to fall from the accelerating rod was measured. The speed of the rotarod accelerated from 4 to 40 rpm over a 5-min period.

**Limb-clasping reflexes**

A limb-clasping test was conducted as previously described[53]. Mice were suspended by the tail for 15 sec and recorded with an iPad (30 frames/sec). Hind-limb clasping was assessed by the severity: 0, normal limb-extension; 1, one hind-limb was retracted; 2, both hind-limbs were retracted. The limb-clasping severity was scored for every 10 frames and total scores for 15 sec (450 frames) were calculated.

**Hot plate test**

Mice were placed on a 55.0 (± 0.3) ºC hot plate (Columbus Instruments, Columbus, OH). Latency to the first withdrawal of paws from the hot plate was measured.

**Startle response/prepulse inhibition test**

A startle response/prepulse inhibition test was conducted as previously described[54]. A startle reflex measurement system (O'Hara & Co.) was used. Mice were placed in the plastic cylinder where they were left undisturbed for 10 min for habituation just before the test. In the test, 40 ms duration of white noise was used as the startle stimulus. The intensity of the startle stimulus was 110 or 120 dB. The background noise level was 70 dB. The prepulse sound, 74 or 78 dB, was presented 100 ms before the startle stimulus. A test session consisted of six trial types, i.e., two types for the startle stimulus-only trials (110, 120 dB), and four types for the prepulse inhibition trials (74-110, 78-110, 74-120, 78-120 dB). The average inter-trial interval was 15 sec (range 10-20 sec). The startle response was recorded for 140 ms starting with the onset of the prepulse stimulus, and the peak startle amplitude during the 140 ms was used as the dependent variable.

**Light/dark transition test**

A light/dark transition test was conducted as previously described[55]. Mice were placed into the dark chamber and 3 sec later the door was opened. The mice were allowed to move freely between the two chambers with the door open for 10 min. The total number of transitions between chambers, latency to first entry to the light chamber, time spent in each chamber and total distance traveled were recorded.

**Elevated plus maze test**

An elevated plus maze test was conducted as previously described[56]. The number of entries into an arm, time spent in the open arm and the enclosed arm were measured for 10 min.

**Marble burying test**

A marble burying test was conducted as previously described[57,58]. A cage (40 × 25 × 18 cm) was filled 5 cm deep with ALPHA-dri bedding (Shepherd Specialty Papers, Watertown, TN). 20 glass marbles (12.5 mm diameter, dark green) were arranged in a 4 × 5 grid on the bedding. Mice were placed in the cage and allowed to freely explore for 30 min. The number of buried marbles covered by the bedding up to 1/2 of their depth was counted.

**Porsolt forced swim test**

Plastic cylinders (22 cm height, 12 cm diameter) were filled up with hypochlorous water (23 ºC) up to a height of 7.5 cm. Mice were placed into the cylinders, and behaviors were recorded for 10 min.

**Tail suspension test**

Mice were suspended by their tails 30 cm above the floor with adhesive tape and behaviors were recorded for 10 min.

**Cued and contextual fear conditioning**

A cued and contextual fear conditioning test was conducted as previously described[59]. Mice were placed in a square test chamber with a metal grid floor. A 55 dB white noise was presented as a conditioned stimulus (CS) for 30 sec, and a 0.3 mA foot shock was given to the mice as an unconditioned stimulus (US) during the last 2 sec of the white noise. Each mouse received the three CS-US pairings with 2 min intervals in a conditioning session. At 24 hours and at 29 days after the conditioning session, contextual testing and cued testing with altered context was performed. In the contextual testing, each mouse was placed in the same test chamber for 5 min without CS or US. In the cued testing with altered context, mice were placed in a novel triangular test chamber for 6 min. The CS and the US were not presented in the first 3 min, and the CS (a 55 dB white noise) was presented for the last 3 min. Freezing was measured during the conditioning, contextual testing, and cued testing sessions.

**Supplementary References**


51. Koshimizu, H., Takao, K., Matozaki, T., Ohnishi, H. & Miyakawa, T. Comprehensive behavioral analysis of cluster of differentiation in 47 knockout mice. *PLoS one* **9,** e89584 (2014).
52. Sashindranath, M., Daglas, M. & Medcalf, R. L. Evaluation of gait impairment in mice subjected to craniotomy and traumatic brain injury. *Behav. Brain Res.* **286,** 33-38 (2015).
53. Lieu, C. A., Chinta, S. J., Rane, A. & Andersen, J. K. Age-related behavioral phenotype of an astrocytic monoamine oxidase-B transgenic mouse model of Parkinson's disease. *PLoS one* **8,** e54200 (2013).
54. Matsuo, N et al. Behavioral profiles of three C57BL/6 substrains. *Front. Behav. Neurosci.* **4,** 29 (2010).
55. Takao, K. & Miyakawa, T. Light/dark transition test for mice. *J. Vis. Exp.* **1,** e104 (2006).
56. Komada, M., Takao, K. & Miyakawa, T. Elevated plus maze for mice. *J. Vis. Exp.* **22,** e1088 (2008).
57. Thomas, A. et al. Marble burying reflects a repetitive and perseverative behavior more than novelty-induced anxiety. *Psychopharmacology.* **204,** 361-373 (2009).
58. Kedia, S. & Chattarji, S. Marble burying as a test of the delayed anxiogenic effects of acute immobilization stress in mice. *J. Neurosci. Methods* **233,** 150-154 (2014).
59. Shoji, H., Takao, K., Hattori, S. & Miyakawa, T. Contextual and cued fear conditioning test using a video analyzing system in mice. *J. Vis. Exp.* **85,** e50871 (2014).
